# Effects of Ankle Orthoses, Taping, and Insoles on Postural Stability of Individuals with Chronic Ankle Instability: A Systematic Review

**DOI:** 10.3390/healthcare11182570

**Published:** 2023-09-18

**Authors:** Yunqi Tang, Peiyao Liang, Jingwen Pan, Cui Zhang, Hui Ren, Shizhe Cheng, Pui Wah Kong

**Affiliations:** 1 College of Art and Design, Shaanxi University of Science and Technology, Xi’an 710021, China; tangyunqi@sust.edu.cn (Y.T.); 211011033@sust.edu.cn (P.L.); 211011025@sust.edu.cn (H.R.); 211012049@sust.edu.cn (S.C.); 2 Physical Education and Sports Science Academic Group, National Institute of Education, Nanyang Technological University, Singapore 637616, Singapore; nie173748@e.ntu.edu.sg; 3 Rehabilitation Research Institute of Singapore, Nanyang Technological University, Singapore 308232, Singapore; 4 Sport Biomechanics Laboratory, Shandong Institute of Sports Science, Jinan 250014, China; zhangcui@sdpei.edu.cn; 5 Graduate School, Shandong Physical Education University, Jinan 250014, China

**Keywords:** injury, rehabilitation, chronic ankle instability, orthoses, taping, insoles

## Abstract

Chronic ankle instability (CAI) is a prevalent condition characterized by recurring instances of the ankle giving way and persistent symptoms, including pain and diminished function. Foot and ankle external supports are commonly used in clinical practice and research for treating CAI. This systematic review aimed to assess the effects of foot and ankle external supports on the postural stability of individuals with CAI to guide clinical practice and inform future research. A comprehensive search was conducted in PubMed, Web of Science, Scopus, and Google Scholar databases from 1 January 2012 to 1 November 2022. Eighteen studies involving individuals with CAI were chosen in this systematic review. The quality of the included studies and risk of bias were assessed using Cochrane Collaboration’s tool for randomized controlled trials, the Newcastle–Ottawa Scale for case–control studies, and the DELPHl-list for crossover trial studies. The external supports included in this review were ankle orthoses (elastic, semi-rigid, and active orthoses), taping (kinesiotaping and fibular reposition taping), and insoles (textured and supportive insoles). The outcome measures included static and dynamic postural stability tests, such as the single-leg stance test, star excursion balance test, Y-balance test, single-leg landing test, lateral jump test, walking test, and running test. The results showed that elastic orthoses, Kinesiotaping, and textured insoles demonstrated potential benefits in improving postural stability in individuals with CAI. Elastic orthoses decreased ankle joint motion variability, kinesiotaping facilitated cutaneous receptors and proprioceptive feedback, while textured insoles increased tactile stimulation and foot position awareness. However, the effects of semi-rigid orthoses, fibular reposition taping, and arch support insoles were inconsistent across studies. Future research should explore the long-term effects of these external supports, analyze the effects of different characteristics and combinations of supports, and employ standardized outcome measures and testing protocols for assessing postural stability.

## 1. Introduction

Postural stability is the ability to maintain or re-establish the center of mass (COM) within the base of support during both static and dynamic tasks [1]. Postural stability is essential for performing daily activities and preventing falls and injuries, especially for individuals with chronic ankle instability (CAI). CAI is a prevalent condition characterized by recurring instances of the ankle giving way and persistent symptoms, such as pain and diminished function [2]. CAI can impair postural stability by affecting the mechanical, sensorimotor, and psychological factors that contribute to balance control. Therefore, restoring postural stability is a primary objective in CAI treatment [3].

Various interventions have been proposed to improve postural stability in individuals with CAI, such as surgical and conservative approaches [4]. Surgical intervention involves repairing or reconstructing the damaged ligaments to restore mechanical stability to the ankle joint [5]. However, surgical procedures are typically reserved for patients who have not responded to conservative treatment or exhibit severe mechanical instability [6]. Conservative treatment involves various modalities, such as proprioceptive exercise [7], manual therapy [8], neuromuscular electrical stimulation [9], bracing [10], taping [11], and orthoses [12]. The proprioceptive exercise involves performing balance training tasks on various surfaces or devices to enhance ankle joint position sense and muscle activation [7]. Manual therapy involves applying passive movements or manipulations to the ankle joint or surrounding soft tissues to improve joint mobility and reduce pain [8]. Neuromuscular electrical stimulation involves applying electrical currents to stimulate muscle contraction or sensory nerve endings to modulate pain [9]. Previous systematic reviews and meta-analyses have reported that these interventions can improve postural stability, reduce pain, and increase function in individuals with CAI [13]. However, there is still a lack of consensus on the optimal type, intensity, frequency, and duration of these interventions for CAI.

Among the conservative modalities, foot and ankle external supports are widely used in clinical practice and research for treating CAI [14,15,16]. These external supports include different types of ankle orthoses (e.g., elastic, semi-rigid, and active orthoses), taping techniques (e.g., kinesiotaping and fibular reposition taping (FRT)), and insoles (e.g., textured and supportive insoles). The mechanisms of these external supports may involve limiting excessive ankle joint range of motion, stimulating the cutaneous mechanoreceptors and proprioceptors around the ankle joint, improving proprioceptive feedback and neuromuscular control, reducing fatigue and pain, and enhancing confidence and performance.

Postural stability can be measured using various methods that assess different aspects of balance performance. These methods include force platforms that measure center of pressure (COP) parameters [17], functional tests that measure lower extremity reach distance or jump distance [18], motion analysis systems that measure joint angles or angular variability [19], and electromyography systems that measure muscle reaction time or activation [20]. These methods have different levels of reliability, validity, and sensitivity in detecting postural stability impairments or changes in individuals with CAI [21]. Generally, force platforms and motion analysis systems are more objective and precise than functional tests and electromyography systems [22]. On the other hand, functional tests and electromyography systems are more practical and ecological than laboratory equipment [23]. Postural stability can also be classified into static and dynamic stability according to the task condition. Static stability refers to maintaining or re-establishing COM within the base of support during quiet standing or minimal movement tasks [24]. Dynamic stability refers to maintaining or re-establishing COM within the base of support during movement tasks that involve changes in COM position or velocity [25]. Static and dynamic stability may reflect different aspects of balance control and require different sensorimotor mechanisms [26].

Previous studies have reported inconsistent or conflicting results regarding different types or combinations of external supports [27,28]. Moreover, most studies have focused on the immediate effects of external supports on postural stability, while few studies have investigated their long-term effects [27,29].The effects of foot and ankle external supports on postural stability specific to individuals with CAI remain unclear. Given the importance of postural stability for individuals with CAI and the variety of foot and ankle external supports available for its improvement, a systematic review is warranted to summarize and evaluate the current evidence on this topic. This study aimed to investigate the effects of external foot and ankle support on the postural stability of individuals with CAI by identifying and appraising relevant studies, comparing and synthesizing their results, and discussing the quality and limitations of the existing evidence. Findings from this systematic review provide valuable information to guide future clinical and practical research.

## 2. Methods

This study was registered with the International Prospective Register of Systematic Reviews (Prospero ID: CRD42023458770).

### 2.1. Search Strategy

A comprehensive search was conducted on PubMed, Web of Science, Scopus, and Google Scholar from 1 January 2012 to 1 November 2022. The search strategy was developed with a combination of Medical Subject Headings and free-text terms. The search strategy was as follows: (“ankle instability” OR “chronic ankle instability”) AND (“tape” OR “taping” OR “supports” OR “orthoses” OR “insole” OR “brace”). The reference lists of the retrieved articles were also screened for additional relevant studies.

### 2.2. Eligibility Criteria

Studies were included if they met the following criteria: (a) involved patients diagnosed with CAI, (b) utilized foot and ankle external supports as interventions, (c) employed a quantitative research design, which we defined as a study that collects and analyses numerical data to understand the phenomena of interest, (d) measured postural stability outcomes, and (e) were written in English. Studies were excluded if they (a) contained confounding factors other than foot and ankle external supports or (b) were qualitative studies, case studies, reviews, dissertations, or conference papers.

Two reviewers independently screened the titles and abstracts of the retrieved records and assessed the full texts of potentially eligible studies. Any disagreements were resolved through discussion or, if necessary, consultation with a third reviewer.

### 2.3. Study Quality Assessment and Risk of Bias

The quality and risk of bias of the included studies were assessed using different evaluation tools according to their research designs. For randomized controlled trials (RCTs), the Cochrane Collaboration’s tool for assessing risk of bias was used [30]. This tool considered the following domains: sequence generation, allocation concealment, blinding of participants, personnel, and outcome assessors, incomplete outcome data, selective reporting, and other biases. Each domain was judged to have a low, unclear, or high risk of bias. For case–control trials, the Newcastle–Ottawa Scale (NOS) was used [31]. This scale included four items (four points) for the selection of study subjects, one item (two points) for comparability between groups, and three items (three points) for the measurement of outcomes, with a total score of nine points. A score of six or more indicated a high-quality study, while a score of less than six indicated a low-quality study. For repeated measures designs, an adapted version of the DELPHI list was used [32]. This list had scores ranging from three (low quality) to nine (high quality). Studies with a score below three were excluded.

The methodological quality and risk of bias of the included studies were independently assessed by two reviewers. Any reviewer disagreements were resolved through discussion or consultation with a third reviewer. The interrater reliability of the quality assessment scores between the reviewers was measured using Cohen’s kappa [33]. A weighted kappa with linear weights was used because this approach could account for the ordinal nature of the scores. Substantial agreement between the reviewers was indicated by a kappa value of 0.793 (*p* < 0.001).

### 2.4. Data Extraction

Data extraction was performed using a standardized form that included information on the study design, participants and sample size, criteria for CAI, types of foot and ankle external supports, intervention time, and test condition. The primary outcome of interest was postural stability, which was assessed using various static and dynamic tests and indicators.

### 2.5. Data Synthesis

Meta-analysis was not performed in this systematic review due to the high heterogeneity of the included studies regarding the types and characteristics of external supports, the intervention duration, the outcome measures, and the testing protocols. Therefore, a qualitative synthesis of the results was conducted instead.

## 3. Results

### 3.1. Overview of the Included Studies

**Figure 1:**
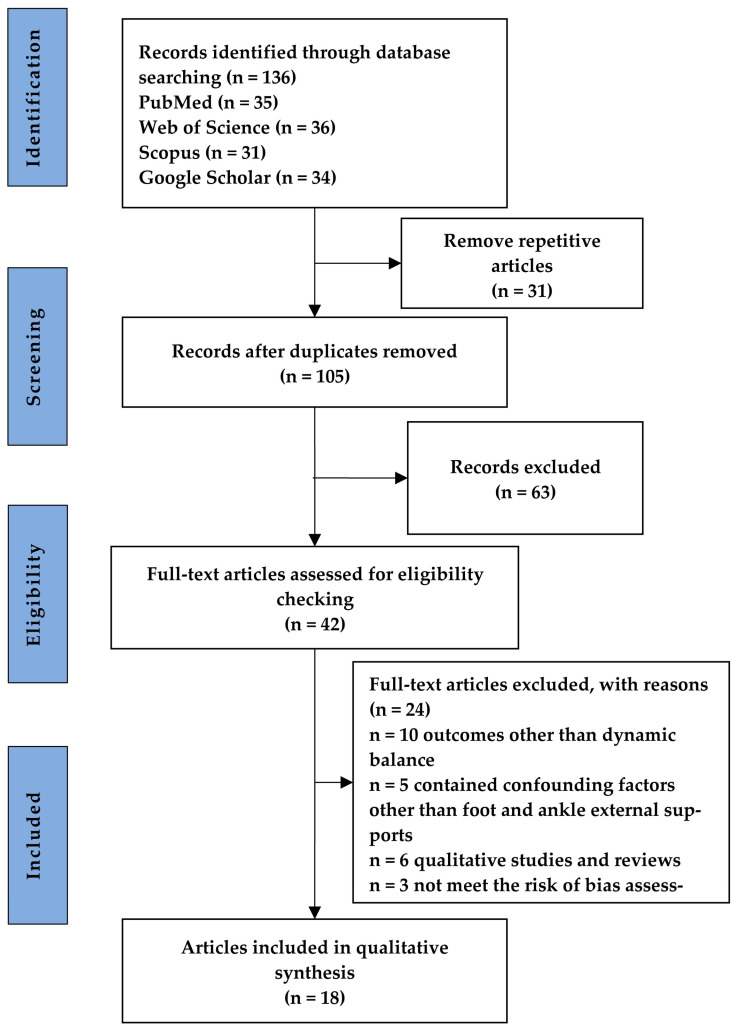
Flow chart for the literature review process.

The procedures of the literature search and selection process are depicted in Figure 1. After the database search, 136 articles were initially identified. Duplicates amounting to 31 articles were subsequently removed. Articles failing to meet the title and keyword evaluation criteria, numbering 63, were excluded. Additionally, 24 articles not meeting the established criteria were discarded. As a result, 18 articles were incorporated into this systematic review (Figure 1).

The reviewed articles involved individuals with CAI, with a total of 497 participants. Five studies also included healthy controls, with a total of 79 participants. Both male and female subjects were included in 13 studies [10,28,34,35,36,37,38,39,40,41,42,43,44], and 5 studies did not report the gender of the subjects [11,45,46,47,48]. The subjects were all young adults aged between 18 and 30 years. The time from the first sprain to the test was at least 12 months in 9 studies [10,11,35,36,39,40,42,47], the first sprain occurred within 12 months in one study [34], and the first sprain occurred within 6 months in one study [45]. However, seven studies did not report the time from the first sprain to the test for the participants [28,37,38,41,43,44,48]. The participants were diagnosed with CAI based on the Cumberland Ankle Instability Tool (CAIT) score ≤24, although a more valid diagnostic criterion is a score ≤21.5 [49]. Seven studies reported that the CAIT score was lower than 21.5, while two studies did not report it. Another inclusion criterion for CAI was the Foot and Ankle Ability Measure (FAAM) score <85%. Six studies reported that the FAAM score ranged from 73.2% to 84.6% [11,36,37,40,43,46], while one study [41] did not provide FAAM scores. Four studies only described the inclusion criteria without using any scales [10,35,38,45]. One study [28] used a score of ≤90% on the Foot and Ankle Disability Index (FADI) as an inclusion criterion for CAI.

The included studies involved different types of foot and ankle external supports, such as ankle orthoses (Table 1), taping (Table 2), and insoles (Table 3). The ankle orthoses included elastic, semi-rigid, and active orthoses; the taping included kinesiotaping and FRT. The insoles included textured insoles and supportive insoles. The intervention duration ranged from immediate to four weeks. The outcome measures included static and dynamic postural stability tests, such as the single-leg stance test (SLST), star excursion balance test (SEBT), Y-balance test (YBT), single-leg landing test, lateral jump test, and walking and running tests. The primary outcome indicators were center of pressure (COP) parameters, lower extremity reach distance, joint angles and angular variability, jump height and distance, time to boundary, time to stabilization, muscle reaction time, and a composite measure of postural stability.

### 3.2. Risk of Individual Studies

The risk of bias in five RCT studies [11,38,44,46,48] was assessed using Cochrane Collaboration’s tool (Table 4). These studies had a low risk of bias in terms of “Randomization”, “Allocation concealment”, “Incomplete outcome data”, and “Selective reporting”. However, the domains related to blinding were considered to have a high risk of bias.

The quality of five case-control studies [34,35,36,37,45] was evaluated using the NOS Scale (Table 5). All the included studies scored more than 6 points, indicating high quality. The average score was 8.2 points. The main item that lost points was “no response rate”. These studies scored high in “selection of study subjects” and “comparability between groups”.

Eight studies (crossover trial [28,39,41,42,43,47], n = 6; controlled laboratory study [10,40], n = 2) were appraised using the DELPHI list (Table 6). The average score was 4.25 points. The reason for the low score was that almost all studies did not blind the assessors and therapists and did not provide an intention-to-treat analysis.

## 4. Discussion

This systematic review summarized the effects of different types of foot and ankle external supports on the postural stability of individuals with CAI. The external supports included in this review were ankle orthoses, taping, and insoles. It was found that elastic orthoses, kinesiotaping, and textured insoles could improve postural stability in individuals with CAI. However, the effects of semi-rigid orthoses, FRT, and arch support insoles were inconclusive.

### 4.1. Effect of Ankle Orthoses on Postural Stability of Individuals with CAI

The reviewed literature included seven articles that evaluated the effects of ankle orthoses on postural stability in individuals with CAI. The results are inconclusive regarding the effects of semi-rigid orthoses. However, the majority of the studies found that elastic orthoses were effective in improving postural stability in individuals with CAI.

Elastic orthoses are ankle protection products that mainly consist of elastic materials, which aim to wrap and protect the ankle joint and improve its stability. Four studies in the reviewed literature examined the effect of elastic orthoses on individuals with CAI. Three of them reported that elastic orthoses enhanced postural stability in individuals with CAI, as indicated by reduced errors in the SLST, increased single-leg standing time, increased reach distances in the YBT, and reduced ankle joint sagittal plane angle variability during walking [34]. However, one study found no effects, as they observed no differences in the oscillation rate of COP and reach distances in SEBT before and after wearing elastic orthoses. These effects were observed immediately after wearing the orthoses or after a four-week intervention. This discrepancy may be attributed to the differences in the materials of the elastic orthoses. The ankle orthoses used in the study consisted of thick, nonelastic, soft materials and elastic figure-eight shape bandages, while the ankle orthoses used in other studies were made of elastic fabrics combined with nonrigid support or figure-eight bandages. Elastic materials have stronger wrapping capabilities than nonelastic materials, which could improve the dynamic and static posture control ability of individuals with CAI. Elastic orthoses possibly improve proprioceptive input by stimulating the skin mechanoreceptors around the ankle joint. This stimulation may compensate for sensory deficits and enhance the postural control abilities of individuals with CAI [50]. Future studies should investigate the optimal characteristics of elastic orthoses, such as material, size, shape, and tension, and compare their effects with other interventions, such as exercise or manual therapy.

Semi-rigid orthoses are ankle braces made out of plastic, metal, or other rigid materials to support the ankle joint. The effects of semi-rigid orthoses on postural stability in individuals with CAI were inconsistent across studies. Two studies reported that wearing semi-rigid orthoses with U-shaped plastic support reduced COP sway and increased reach distances in the SEBT in both forward and lateral directions. Similarly, another study using semi-rigid orthoses consisting of a brace and an insole connected by movable plastic splints reported increased SEBT reach distances after wearing the ankle orthoses. However, one study [45] using semi-rigid orthoses with spring steel bars and figure-eight shape bandages found no significant changes in SEBT reach distances. These discrepancies in the results may be attributed to the differences in the designs and structures of the semi-rigid orthoses and the limitations in motion imposed by the rigid support. Therefore, further research is needed to determine the effects of semi-rigid orthoses on individuals with CAI and to identify any structural differences that may affect their effects.

Recently, active orthoses have attracted attention as they are a novel form of ankle support for individuals with CAI. Two studies used the same type of active orthoses, which consist of a calf sleeve and an insole connected by laces embedded in an energy absorption system on the lateral side of the sleeve. The system contains an exchangeable module that can autonomously adjust the laces according to joint motion [10,35]. Studies have shown that active orthoses could reduce mediolateral COP sway during SLST for individuals with CAI and reduce the ankle inversion angle during sudden inversion disruptions [10]. These findings suggest that active orthoses can effectively control excessive joint activity in the ankle. However, research on active orthoses is limited, and the effect on postural stability during movements such as walking and running is not well understood. Further investigation is needed to better understand the potential benefits of these ankle orthoses for individuals with CAI.

### 4.2. Effect of Taping on Postural Stability of Individuals with CAI

The review included seven studies that investigated the effects of taping on postural stability in individuals with CAI, which mainly focused on kinesiotaping and FRT. Some studies have shown that kinesiotaping could improve postural stability in individuals with CAI, while the effects of FRT are still unclear [41,46,47].

Kinesiotaping is a technique that involves applying elastic tape to the ankle joint in a neutral position, covering the malleoli and heel with moderate tension. The amount of tape stretching varied in the literature, ranging from 50% to 75% or not being reported at all [39,40,46,48]. These effects were observed immediately after applying the tape [39,40,46] or after a seven-day intervention [48]. Four studies demonstrated that kinesiotaping can positively affect individuals with CAI by improving their postural stability and joint kinematics [39,40,46,48]. These effects include an increase in lower limb reach distance during the SEBT, a decrease in jump distance during the single-leg hop test, an increase in composite score following a seven-day intervention, and a reduction in ankle dorsiflexion and inversion during walking and running. The underlying mechanism of kinesiotaping is believed to involve providing both mechanical support and sensory stimulation to the ankle joint. This dual action may contribute to the prevention of excessive joint motion and the enhancement of proprioceptive feedback. Future studies should investigate the optimal application methods of kinesiotaping, such as direction, length, width, tension, and pattern of tape placement, and compare their effects with other taping techniques or orthoses.

FRT is a technique that involves applying tape to the ankle joint in a neutral position while displacing the distal fibula posteriorly and superiorly. The taping procedure starts from the lateral side of the ankle joint, with the tape being slightly obliquely pulled over the Achilles tendon and secured above the starting position [41,46,47]. Two studies compared the effects of a tensioned FRT and a non-tensioned FRT with a similar wrapping form but without repositioning the fibula or stretching the tape. The results showed that FRT could increase the lateral SEAT reach distance and improve dynamic stability in individuals with CAI compared to no tape or non-tensioned tape. Moreover, the response time of the fibularis longus muscle during running was shortened when wearing FRT, which may be due to the improved proprioception and increased power generation of ankle muscles. However, two studies [46,47] revealed no significant difference in the frequency of the non-affected leg touching the ground and COP velocity during single-leg standing among the FRT, untaped, and non-tensioned tape groups. This indicated that tape tension and fibular reposition peroneal retaping had limited effects on the static stability of individuals with CAI [24,27]. This outcome is inconsistent with the dynamic stability result, which may explain the varied intensities of the testing movements. During single-leg standing, the ankle joint adopts a fixed posture, and the pulling tension of the FRT may not be strong enough to stimulate the skin receptors and affect external posture control. Currently, research on FRT tape is limited, and its effect is unclear. Therefore, further investigation into the effect of FRT tape on individuals with CAI and the comparison of various taping methods and tape tensions are necessary.

### 4.3. Effect of Insoles on Postural Stability of Individuals with CAI

The review identified five studies that examined the effects of insoles on postural stability in individuals with CAI [28,36,42,43,44]. The results showed that textured insoles positively improved postural stability in individuals with CAI, while the effects of arch support insoles were inconsistent.

Textured insoles are designed with raised patterns on their surface, such as hemispherical bumps or grid-like textures [28,42,43]. Research studies have demonstrated that textured insoles, compared to smooth-surfaced insoles, have several beneficial effects on individuals with CAI. These effects were observed immediately after wearing the insoles or after a two-week intervention. These effects include a decrease in the medial-lateral COP time-to-boundary variability during quiet standing, an increase in lower limb reach distance during the SEBT, and a decrease in sagittal plane ankle motion variability during walking [28,42,43]. The underlying mechanism of textured insoles is believed to involve enhancing sensory input from the plantar surface by stimulating mechanoreceptors and augmenting cutaneous feedback. The raised patterns on the insoles provide additional tactile stimulation, which may improve the individual’s perception of foot position and movement. This enhanced sensory input can improve postural stability, increase reach distance, and reduce ankle motion variability in individuals with CAI. Future studies should investigate the optimal characteristics of textured insoles, such as texture type, size, shape, and density, and compare their effects with other types of insoles or orthoses.

Arch support insoles feature a raised area at the arch region and a heel cup with a height of 2–3 cm [36,42,43,44]. Some studies have shown that arch support insoles can improve postural stability in individuals with CAI [42,44]. These improvements may include a decrease in COP envelope area during SLST or an increase in lower limb reach distance during SEBT compared to flat insoles [42,44]. However, other studies indicated that arch support insoles did not significantly affect postural stability in individuals with CAI [36,43]. In these studies, there was a lack of reduction in frontal plane ankle motion variability during walking, COP sway, or lower limb reach distance during the single-leg stance test or SEBT compared to flat insoles or no insoles.

The inconsistent results may be attributed to participants’ differences in the pathophysiology of CAI, which can be determined by the complex interplay of ligament laxity, proprioceptive deficit, and muscle control [2]. Arch support insoles may enhance sensory input by increasing contact area or reduce joint control burden by shifting pressure to the medial arch region [51]. However, compared to the more muscular plantar stimulation provided by textured insoles or the mechanical stabilization offered by ankle orthoses, the effects of arch support insoles alone may be limited. Future studies should further explore the effects of arch support insoles and investigate the potential benefits of combining arch support with other types of external supports.

### 4.4. Limitations

This systematic review has some limitations that should be acknowledged. First, we did not perform a meta-analysis to quantitatively synthesize the results or estimate the overall effect size of the external supports. This was due to the high heterogeneity of the included studies, which limited the validity and reliability of the meta-analysis. However, a meta-analysis would have provided more robust and precise evidence for the effects of external supports on postural stability in individuals with CAI. In addition, we did not consider the potential moderators or mediators of the effects of external supports, such as sex, age, and severity of CAI. These factors may confound or moderate the effects of external supports in different subgroups among individuals with CAI. Therefore, our results may not be generalizable or applicable to all individuals with CAI. Another limitation of this systematic review is the heterogeneity of the stability measurement methods across studies. They do not assess the same aspect of stability, as some are dynamic, and others are static. This may compromise the comparability and validity of our results.

## 5. Conclusions

This systematic review provides a comprehensive overview of the effects of various foot and ankle external supports on the postural stability of individuals with CAI. The results showed that elastic orthoses, kinesiotaping, and textured insoles demonstrate potential benefits in improving postural stability in individuals with CAI. Physicians, physiotherapists, podiatrists, and other healthcare professionals can consider these strategies to improve the stability of their patients with unstable ankles. However, the effects of semi-rigid orthoses, FRT, and arch support insoles remain inconclusive. To advance the current knowledge, future studies should investigate the long-term effects of external supports on postural stability in individuals with CAI. Additionally, it is crucial to analyze the effects of different characteristics and combinations of external supports to determine the most effective interventions. Moreover, standardized outcome measures and testing protocols for postural stability assessment would facilitate the comparability and integration of research findings.

## Figures and Tables

**Table 1:** Characteristics of the included studies investigating the effects of ankle orthoses in individuals with chronic ankle instability (CAI).

References	Study Design	Participants (Number; Sex; Mean Age (Years))	The First Sprain Time	The Score of Criteria for CAI	Types of Ankle Orthoses	Intervention Time	Test Condition
Stotz et al., 2021 [34]	Case–control	CAI (n = 14; M/F: 6/8; 23.0 ± 3.2) Healthy (n = 13, M/F: 11/2; 26.3 ± 3.7)	within 12 months	CAIT: 17.2 ± 4.1	Elastic orthoses	Immediate effects	Running
Hassanpour et al., 2020 [45]	Case–control	CAI (n = 15; NR; 25 ± 5.32) Healthy (n = 15; NR; 23.7 ± 4.69)	within 6 months	NR	Elastic orthoses, Semi-rigid orthoses	Immediate effects	BBS, SEBT
Hadadi et al., 2020 [11]	RCT	CAI (n = 60; NR; 24.57 ± 1.64)	Over 12 months	CAIT: 12.1 ± 5.2 FAAM: 73.6 ± 8.8%	Kinesiotaping, Elastic orthoses, Semi-rigid orthoses	Four weeks	SLST, SEBT, SLHT
Raffalt et al., 2019 [35]	Case–control	CAI (n = 16; M/F: 9/7; 30.9 ± 4.7) Healthy (n = 9; M/F: 7/2; 29.3 ± 4.5)	Over 12 months	NR	Active orthoses	Immediate effects	SLST
Hadadi et al., 2019 [36]	Case–control	CAI (n = 22; M/F: 10/12; 22.7 ± 2.6) Healthy (n = 22; M/F: 10/12; 23.1 ± 2.9)	Over 12 months	CAIT: 16.8 ± 6.2 FAAM: 81.4 ± 2.7%	Semi-rigid orthoses, Support insole, Soft orthoses	Immediate effects	SLST, SEBT SLST, SEBT
Agres et al., 2019 [10]	Controlled laboratory study	AI (n = 16, M/F: 9/7; 30.9 ± 64.7)	Over 12 months	NR	Active orthoses	Immediate effects	SLDL IPT
Hadadi et al., 2017 [37]	Case–control	CAI (n = 20 n = 22; M/F: 10/10; 21.9 ± 2.4) Healthy (n = 20; M/F: 10/10; 22.8 ± 3.2)	NR	CAIT: 16.2 ± 5.3 FAAM: 84.2 ± 7.5%	Semi-rigid orthoses	Immediate effects	SLST

Abbreviations: RCT, randomized controlled trial; CAI, chronic ankle instability; M, male; F, female; CAIT, Cumberland Ankle Instability Tool; FAAM, Foot and Ankle Ability Measure; BBS, Biodex Balance System; SLST, single leg stance test; SEBT, Star Excursion Balance Test; SLHT, single-limb hopping test; SLDL, single-legged drop landing; IPT, inversion plate test; NR, not reported.

**Table 2:** Characteristics of the included studies investigating the effects of taping in individuals with CAI.

References	Study Design	Participants (Number; Sex; Mean Age (Years))	The First Sprain Time	The Score of Criteria for CAI	Types of Taping	Intervention Time	Test Condition
Hadadi et al., 2020 [46]	RCT	CAI (n = 60; NR; 24.57 ± 1.64)	Over 12 months	CAIT: 12.1 ± 5.2 FAAM: 73.6 ± 8.8%	FRT, Placebo taping	Two weeks	SLST, SEBT, SLHT
Alawna et al., 2020 [38]	RCT	CAI (n = 100; Taping group: M/F: 18/15, 22.25 ± 2.96; Bandaging group: M/F: 19/14, 23.56 ± 4.25; Placebo group: M/F: 19/15, 22.95 ± 3.24)	NR	NR	Ankle rigid taping, Ankle bandaging, and Placebo taping	Two months	YBT, Vertical jump height measurements
Yen et al., 2018 [39]	Crossover trialCrossover trial	CAI (n = 20; M/F: 15/5; 22.9 ± 1.6)	Over 12 months	CAIT: NR	Kinesiotaping, Athletic taping	Immediate effects	Walking
Alves et al., 2018 [47]	Crossover trial	CAI (n = 16; NR; 21.5 ± 2.8)	Over 12 months	CAIT: 19.4 ± 5.4	FRT, Placebo taping	Immediate effects	SLST, Functional performance test (figure-of-8 hop test, Lateral hop test)
de-la-Torre-Domingo et al., 2015 [48]	RCT	CAI (n = 30; Kinesiotaping group: NR, 18.87 ± 1.81; Placebo group: NR, 20.07 ± 1.58)	NR	CAIT: NR	Kinesiotaping, Placebo taping	Seven days	SOT
Chinn et al., 2014 [40]	Controlled laboratory study	CAI (n = 15; M/F: 8/7; 26.9 ± 6.8)	Over 12 months	FAAM: 75.8 ± 13.3%	Kinesiotaping,	Immediate effects	Walking, Running
Wheeler et al., 2013 [41]	Crossover trial	CAI (n = 23; M/F: 8/15; 23.4 ± 2.5)	NR	FAAM: NR	FRT, Placebo taping	Immediate effects	SEBT

Abbreviations: RCT, Randomized Controlled Trial; M, male; F, female; CAI, Chronic Ankle Instability; CAIT, Cumberland Ankle Instability tool; FAAM, Foot and Ankle Ability Measure; FRT, Fibular Reposition Taping; SLST, Single Leg Stance Test; SEBT, Star Excursion Balance Test; SLHT, Single-limb Hopping Test; YBT, Y Balance Test; SOT, Sensory Organization Test; NR, Not Reported.

**Table 3:** Characteristics of the included studies investigating the effects of insoles in individuals with CAI.

References	Study Design	Participants (Number; Sex; Mean Age (Years))	The First Sprain Time	The Score of Criteria for CAI	Types of Insole	Intervention Time	Test Conditions
Abbasi et al., 2019 [42]	Crossover trial	CAI (n = 30; M/F: 13/17; 22.3 ± 2.7)	Over 12 months	CAIT: 18.3 ± 4.9	Custom-molded with textured surface insole, Custom-molded insole, Prefabricated with textured surface insole	Immediate effects	SEBT
Jamali et al., 2019 [43]	Crossover trial	CAI (n = 21; M/F: 11/10; 25.6 ± 4.8)	NR	FAAM ADLS: 73.6 ± 8.8% FAAM Sport: 63.4 ± 16.9%	Flat insole with smooth surface, Prefabricated laterally wedged insole with smooth surface, Flat insole with textured surface, Prefabricated laterally wedged insole with textured surface	Immediate effects	Walking
McKeon et al., 2012 [28]	Crossover trial	CAI (n = 20, M/F: 12/8; 21.5 ± 5.5)	NR	FADI: 82% ± 9%	Textured insole, Smooth insole	Immediate effects	Stance
Hamlyn et al., 2012 [44]	RCT	CAI (n = 40; Support insole group: M/F: 11/9, 20.0 ± 2.3; flat insole group: M/F: 10/10, 20.5 ± 2.1)	NR	CAIT: 15.8 ± 4.3	Support insole, flat insole	Two weeks	SLST

Abbreviations: RCT, randomized controlled trial; CAI, chronic ankle instability; M, male; F, female; CAIT, Cumberland Ankle Instability Tool; FAAM, Foot and Ankle Ability Measure; ADLS: Activities of Daily Living Score; FADI, Foot and Ankle Disability Index; SEBT, Star Excursion Balance Test; SLST, Single Leg Stance Test.

**Table 4:** The Risk of Bias Assessment (Cochrane Collaboration’s tool) of the Selected Studies.

References	Randomization	Allocation Concealment	Blinding of Participants	Blinding of Personnel	Blinding of Outcome Assessors	Incomplete Outcome Data	Selective Reporting
Hadadi et al., 2020a [11]	Low risk	Low risk	Low risk	High risk	Low risk	Low risk	Low risk
Hadadi et al., 2020b [46]	Low risk	Low risk	Low risk	High risk	Low risk	Low risk	Low risk
Alawna and Mohamed, 2020 [38]	Low risk	Low risk	Low risk	High risk	Unclear risk	Low risk	Low risk
de-la-Torre-Domingo et al., 2015 [48]	Low risk	Low risk	Low risk	High risk	Low risk	Low risk	Low risk
Hamlyn et al., 2012 [44]	Low risk	Low risk	High risk	High risk	High risk	Low risk	Low risk

**Table 5:** The Risk of Bias Assessment (Newcastle–Ottawa Scale) of the Selected Studies.

References	Selection				Comparability		Outcome			Score
	Adequate Definition of Cases	Representativeness of Cases	Selection of Controls	Definition of Controls	Comparability on Most Important Factors	Comparability on Other Risk Factors	Assessment of Outcome	Same Method of Ascertainment of Cases and Controls	No Response Rate	
Stotz et al., 2021 [34]	*		*	*	*	*	*	*		7
Hassanpour et al., 2020 [45]	*	*	*	*	*	*		*	*	8
Raffalt et al., 2019 [35]	*	*	*	*	*	*	*	*		8
Hadadi et al., 2019 [36]	*	*	*	*	*	*	*	*	*	9
Hadadi et al., 2017 [37]	*	*	*	*	*	*	*	*	*	9

Note: * means one point.

**Table 6:** The Risk of Bias Assessment (the DELPHl-list) of the Selected Studies.

References	Randomized Allocation	Concealed Allocation	Baseline Simi-Larity	Inclusion Criteria	Blinded Assessors	Blinded Therapists	Blinded Subjects	Point of Vari-Ability	Intention to Treat Analysis	Score
Agres et al., 2019 [10]			*	*				*		3
Yen et al., 2018 [39]	*		*	*				*		4
Alves et al., 2018 [47]	*	*	*	*			*	*		6
Chinn et al., 2014 [40]	*		*	*				*		4
Wheeler et al., 2013 [41]	*			*				*		3
Abbasi et al., 2019 [42]	*		*	*				*		4
Jamali et al., 2019 [43]	*		*	*				*		4
McKeon et al., 2012 [28]	*	*	*	*			*	*		6

Note: * means one point.

## Data Availability

Not applicable.
